# A Proposed Strategy against Obesity: How Government Policy Can Counter the Obesogenic Environment

**DOI:** 10.3390/nu15132910

**Published:** 2023-06-27

**Authors:** Norman J. Temple

**Affiliations:** Centre for Science, Athabasca University, Athabasca, AB T9S 3A3, Canada; normant@athabascau.ca

**Keywords:** health promotion, healthy diets, prevention of obesity, food prices, food advertising, government policy

## Abstract

An epidemic of obesity emerged in the USA in 1976–1980. The epidemic then spread to many other Westernized nations. Many interventions have been carried out with the goal of lowering the prevalence of obesity. These have mostly taken the form of various types of health promotion (i.e., providing people with education, advice, and encouragement). These actions have achieved, at most, only limited success. A strategy with a better chance of success starts with the recognition that the fundamental cause of obesity is that we live in an obesogenic environment. It is therefore necessary to change the environment so that it fosters a generally healthy lifestyle, thereby leading to enhanced health for the population, including improved weight control. A major goal is to increase the intake of healthy foods (especially fruit, vegetables, and whole grains), while decreasing intake of unhealthy foods (especially ultra-processed foods such as sugar). This will require major changes of many government policies. Some of the required policies are as follows. Schools should implement policies that create a healthy environment for children. For example, they should adopt a policy that only foods of high nutritional quality are sold in vending machines or given to students within school meals. Policies need to go well beyond the school setting; a broad strategy is needed that creates a healthy environment for children. Another important policy is the manipulation of food prices in order to shift the diet toward healthy foods. This requires using subsidies to lower the price of healthy foods, while adding a tax to less healthy foods to increase the price. This policy has been implemented in many cities and countries in the form of a tax on sugar-sweetened beverages (SSBs). The advertising of unhealthy foods (including fast-food restaurants) should be banned, especially where children and adolescents are the major target. Such a ban could be extended to a complete ban on all advertising for unhealthy foods, including that directed at adults. The proposed policy measures are likely to be strongly opposed by food corporations.

## 1. Introduction

The obesity epidemic emerged in 1976–1980, first in the USA, and then appeared in many other Westernized countries [1,2]. Surveys reveal that the dramatic growth in obesity in the USA continued among both sexes until as recently as 2016 [3].

Many factors contribute to excess weight gain and obesity. Of particular importance are energy-dense foods that are tasty and relatively cheap. These facilitate an excess intake of energy. Much evidence points to ultra-processed foods (UPFs) as being of particular importance in causing the obesity epidemic [4]. UPFs, in general, have a high energy content and are rich in salt, sugar, and fat. By contrast, they have a low content of dietary fiber and little-to-no whole foods. Common UPFs include white bread, cookies, candy, margarine, and pizza. Sugar, especially when consumed as sugar-sweetened beverages (SSBs), has an especially strong relationship with obesity [4]. 

In addition to diet, many studies suggest that a lack of physical activity is another important factor implicated in obesity. For example, randomized trials indicate that walking helps lower body weight and reduces the amount of visceral body fat [5,6]. 

These findings are of great value in pointing to the lifestyle rules that tell us how obesity can be prevented. The obesity epidemic has generated an enormous effort to find effective ways to reverse this major threat to population health. This is especially the case as obesity significantly increases the risk of other disorders, including type 2 diabetes, hypertension, and several types of cancer [7]. Health professionals have developed various strategies to counter obesity, both in treatment and prevention [8]. However, it is well established that the treatment of obesity has a poor record of success [9], and for that reason, it is widely accepted that primary prevention should be the top priority. 

A great challenge in dealing with obesity is that the world around us seems as if it had been designed to make everyone obese. Consider the following:

- There is an intense marketing of foods that are closely associated with an increased risk of obesity, most notably UPFs [10]. These foods are heavily advertised and occupy a large amount of shelf space in almost every food store, big and small [11]. As a result, nearly everyone living in the Western world is constantly exposed to fattening foods. The avoidance of becoming overweight therefore requires much self-control.
- An unhealthy diet that is dominated by UPFs is significantly cheaper than a healthy diet [12,13]. This means that food prices pressure people—especially less affluent people—to eat a diet that will increase their risk of developing obesity.
- These challenges are especially relevant to children, most of whom have easy access to UPFs. In addition, smart phones, laptops, and smart TVs are extremely common, acting as strong “push factors” toward a sedentary lifestyle.
- Labor-saving devices are ubiquitous. As a result, the requirement for physical activity has been minimized.

These factors compel the conclusion that the fundamental cause of obesity is that the environment in which we live is obesogenic. From this, it follows that the foundation of an anti-obesity strategy entails bold action to change this environment. The first step is the recognition that because many factors work together to cause obesity, a multi-component approach is required. This argument was made in a 2007 report by UK government scientists [14]:

The obesity epidemic cannot be prevented by individual action alone and demands a societal approach. Tackling obesity requires far greater change than anything tried so far, and at multiple levels: personal, family, community and national. Preventing obesity is a societal challenge, similar to climate change. It requires partnership between government, science, business and civil society.

The importance of tackling the obesogenic environment was also emphasized by the World Health Organization in 2016, with a focus on obesity in children [15]. 

## 2. The Limitations of Health Promotion

By the 1970s, it had become well established that lifestyle—diet, tobacco use, exercise—strongly affects the risk of chronic diseases of lifestyle (CDL). These include most forms of cardiovascular disease (including coronary heart disease (CHD), stroke, and hypertension), obesity, type 2 diabetes, and several types of cancer. It is now widely accepted that a healthy lifestyle has an enormous potential for improving population health. 

Inspired by these findings, dozens of health promotion campaigns have been carried out since the 1980s. The most common goal has been the prevention of CVD by targeting the major risk factors. Accordingly, these interventions have attempted to persuade the target population to adopt a healthier lifestyle in the areas of nutrition, exercise, and smoking. The most common approach used in health promotion is health education combined with encouragement to follow a healthier lifestyle. These interventions have been carried out in various settings, most notably within communities (including schools), at worksites, in the offices of family physicians, and via the internet. Improved weight control has often been included as a goal. 

We briefly review the degree of success achieved by a variety of health promotion interventions as the findings provide our best evidence regarding the extent to which a health promotion strategy can achieve success. What applies to health promotion in general probably also applies to similar interventions in the area of weight control.

The findings from health promotion interventions indicate that efforts to persuade people to adopt a healthier lifestyle achieve a mixed degree of success: some interventions have achieved excellent success, while others have achieved little [16]. Overall, a reasonable estimate of the typical reduction in the risk of CVD (including CHD) is about 5–15%. In other words, most people at risk of CDL are unlikely to be affected.

A variation of this strategy is the targeting of subjects at a high risk of disease, most often CHD. In general, this strategy has been more successful than other types of intervention [17]. The obvious explanation for this success is that the subjects of the intervention are likely to be very motivated to make serious change. This is probably the most cost-effective form of intervention [18]. Two randomized controlled trials demonstrated the potential advantages of this strategy. The subjects selected were overweight and had an impaired glucose tolerance; they were therefore at a high risk of type 2 diabetes. Interventions consisting of dietary change and physical activity lowered the estimated risk by about 58% [19,20]. 

## 3. Changes in Lifestyle across the Population: Lessons Learned

Over the past half century, American people have been bombarded with huge amounts of dietary advice, such as TV programs, books, and articles in newspapers. Citizens have easy access to much dietary information from their governments; this includes food guides and food labels. Despite all this effort, the general population has mostly ignored the recommended dietary changes. This confirms the conclusion from the previous section, namely that health promotion is of limited effectiveness.

This is clearly shown by surveys of the American diet: progress has been mostly underwhelming [21]. For example, only 21.5% of Americans eat at least one serving of a whole fruit per day. Similarly, the intake of refined grains is about fivefold greater than that of whole grains. One number encapsulates the problem: UPFs provide nearly 60% of energy intake in both the USA and UK [22,23].

This lack of significant improvement in the American diet is also seen with exercise. Everyone has heard the message countless times to engage in regular physical activity, but barely half of American adults do so [24]. These findings are similar to the low level of exercise reported around 1990 to 2004 [25]. Finally, the obesity epidemic that struck the USA starting in the late 1970s provides compelling evidence that the general population has failed to lead a healthy lifestyle. 

## 4. Why Are People Resistant to Health Promotion?

It is an obvious statement that people prefer to be healthy than sick. Nevertheless, the above evidence clearly demonstrates that the majority of the population is resistant to health education. What is the explanation for this?

Personal behavior is determined by a complicated interaction of multiple factors. A person’s behavior is likely to be shaped by influences such as their education, the type of work they do and how much they are paid, and where they live. Advertising can influence what people want, but actual purchasing decisions are strongly affected by the price of an item and whether a person has enough money to buy it. People are often averse to changing their behavior patterns that they have followed for many years, especially if there is social pressure that opposes the new behavior. Factors in the local environment also affect behavior decisions. Examples include the level of pollution and opportunities for exercise. Similarly, many people live in a “food desert”, where food stores provide only meagre amounts of healthy food. 

Hindsight is 20–20, and this certainly applies to health promotion. It should not come as a surprise that attempts to achieve major improvements in population health by providing health education is doomed to achieve only limited success.

These arguments are highly relevant to the obesity epidemic. The obesogenic environment has a much more powerful impact on eating than the attempts at self-control in order to avoid obesity. This was argued by Cohen and Farley [26], who concluded that “…eating [is] an automatic behavior, as opposed to one that humans can self-regulate”. This led them to the conclusion that “the focus should be less on nutrition education and more on shaping the food environment”.

## 5. A Government Policy Approach to Controlling the Obesity Epidemic

### 5.1. The Case for Government Policies

An alternative strategy for countering the obesity epidemic is interventions carried out by governments. A strategy based on policy approaches, implemented in the form of interventions, has great potential for changing the national diet in the desired direction and thereby improving population health [27]. This will extend to the prevention of obesity. 

Government policies in support of public health have been in widespread use for at least 200 years. This strategy was highly successful in the area of major infectious diseases. For example, the risk of cholera and typhoid was greatly reduced by the provision of safe drinking water and sewage disposal. Many policies have been implemented in recent decades in order to improve health and safety. For example, cars are now fitted with seat belts. Similarly, smoking is now banned in many places. 

The danger to health posed by lead provides a similar lesson, showing the value of government policy. Until the 1970s, this metal was present in gasoline and paint at a level that was dangerous to health. New policies were then implemented in the USA and other countries. This ordered companies to greatly reduce or remove lead from various products. As a result, after about 15 years, the blood lead concentration of American children was only a quarter of the previously recorded levels [28,29]. 

### 5.2. Government Policies and Nutrition

Many policies have been implemented over the decades with the goal of making food safer and more nutritious. For decades, it has been compulsory to “fortify” some foods with particular micronutrients (e.g., the addition of vitamin D to milk, and folic acid and thiamin to grain products). This policy has achieved much success in terms of increasing the intake of micronutrients that are often inadequate in the diet. These policies in the area of nutrition are generally highly cost-effective.

#### 5.2.1. Lowering the Content of Trans Fatty Acids and Salt in Food

It is instructive to look at government policy in the area of lowering the food content of trans fatty acids and compare this with the policy for lowering the food content of salt. Strong evidence revealed that trans fatty acids increase the risk of CHD [30]. Common food sources included hard margarine and baked goods. In response, around the years 2010 to 2018, food manufacturers in the USA and other countries were ordered to remove these fats from foods [31]. 

Salt intake in most countries is excessive [32] and this increases the risk of hypertension [33,34] and cardiovascular disease (CVD) [34,35]. The most effective response to these findings is the implementation of a policy that compels food manufacturers to cut the salt content of food. This would help lower the risk of CVD [36,37]. Alas, such a policy has never been implemented. The UK government implemented a policy based on voluntary action by industry [38]. This led to a fall in salt intake by adults in the UK of about 10% [39], which is far less than the required 30–40% reduction in salt intake. 

The lesson we can learn from these contrasting policies is that an effective strategy for combating the obesity epidemic should emulate the policies used for trans fatty acids (i.e., ordering a reduction in the food content of trans fatty acids) rather than those used in the UK for salt (i.e., voluntary action).

#### 5.2.2. Policies in Schools

A strategy that aims to counter the obesity epidemic must make schools a priority area. There are several reasons for this. First, an unhealthy lifestyle that leads to obesity starts in childhood. Teachers therefore have a responsibility to protect the health of their students by encouraging them to develop a healthy lifestyle. Second, as teachers have a captive audience who are likely to believe what they are told, the classroom is the perfect venue for health promotion. 

However, health promotion in schools can go much further and strive to create a healthy environment. Schools should not only talk the talk, but also walk the walk. This means implementing a policy so that only healthy foods and beverages are available in vending machines. At present, it is common in North America for these machines to stock unhealthy foods and beverages. This policy needs to be extended to meals served at schools. Evidence shows that implementing policies along these lines results in students consuming a healthier diet [40].

#### 5.2.3. Policies Directed at Children

The implementation of policies that create a healthy environment for children should go well beyond the school setting. 

An excellent example of a policy of the type advocated here is called Romp and Chomp, which was carried out during the years 2004 to 2008 [41]. The subjects included 12,000 young children in Australia. The intervention was wide ranging in its approach, and was designed to make the diets healthier and encourage the children to engage in more exercise. The ultimate goal was to reduce the prevalence of obesity. The intervention achieved much success. For example, the investigators made the following observation: “Early-childhood settings in the intervention areas are now places in which fruit, vegetables, and water are promoted and packaged snacks and sweet drinks are restricted or discouraged”.

An especially interesting intervention was carried out in two towns in France over the years 1992 to 2004 [42]. The intervention was at first focused on nutrition education directed at children. This was later expanded to efforts to persuade both adults and children to follow a healthier lifestyle, including engaging in more physical activity. The intervention achieved much success in slowing the rate of excess weight gain in children. Among children aged 5–12, comparison of the two intervention towns with two control towns revealed much lower rates of overweight or obesity in both girls and boys (10.4% vs. 16.0%, 7.4% vs. 19.4%, respectively). 

The limited evidence currently available suggests that the most effective strategy for curbing the obesity epidemic in children consists of policies and action across a broad front. This can potentially achieve a remarkable degree of success. 

A mention is needed of a broad-based program being carried out in Amsterdam [43,44]. The goal is to prevent obesity in children. Known as the Amsterdam Healthy Weight Programme, it is scheduled to run from 2013 to 2033. 

#### 5.2.4. The Potential of Taxes and Subsidies for Improving Diets

Unhealthy foods are generally cheaper than healthier foods [13]. This is especially the case when the cost of food is expressed as cost per unit of energy. For example, obtaining calories from sugar-rich foods costs much less than from fruits and vegetables [12]. Similarly, Belgium researchers reported that the cost of food energy obtained from ultra-processed food is less than half the cost of obtaining it from unprocessed or minimally processed foods (0.55 vs. 1.29 euros per 100 kcal) [45]. These price comparisons point to two conclusions: first, the relatively low cost of an unhealthy diet is an important factor that creates an obesogenic environment, and second, the manipulation of food prices should be seriously considered as a policy approach to combating obesity.

Such a policy approach means lowering the price of healthy foods in comparison with less healthy foods. This can be achieved by providing subsidies for healthy foods and adding a tax on less healthy foods. In order to achieve a significant impact on sales, a change in food prices of about 10% to 15% is needed [46]. Much evidence suggests that this will have a positive impact on the national diet [47] and thereby improve population health.

This concept is based on the well-established inverse relation between price and sales, a phenomenon referred to by economists as price elasticity. This rule has been demonstrated many times in relation to tobacco and alcohol. We see the application of this rule most often in supermarkets when the price of fruit is lowered so that it is sold quickly before it becomes overripe. 

It is important that this policy does not result in excessive price distortions. This can be avoided by the careful regulation of the level of subsidies and taxes. Likewise, the price adjustments can be made revenue-neutral for governments simply by balancing the cost of subsidies with the increased revenue from taxes. 

The one area where this strategy has been put into practice multiple times is in relation to sugar-sweetened beverages (SSBs). A tax has been added to SSBs in various cities in the USA and several dozen other countries. This strategy is effective as consumption is lowered by about 10% after a 10% tax is added to SSBs [48,49]. Other sugar-rich foods, such as chocolate and cookies, should also be seriously considered for such a tax as evidence suggests that this would have a bigger impact than a tax on SSBs [50].

The policy proposed here is likely to have its greatest impact on people living on a low income. Many studies have demonstrated that this section of the population has the least healthy diets. This is inevitable as unhealthy diets are relatively cheap, a point that was noted above. This helps explain the strong association between a low income, poor health, and high rates of obesity. 

Another policy option to help improve the diets eaten by poorer people is to give them coupons that are exchangeable for healthy foods. Coupons of this type are commonly called “food stamps” in the USA. 

#### 5.2.5. A (Partial?) Ban on Food Advertising

The marketing of unhealthy foods is promoted with heavy advertising. This is another contributing factor to the obesogenic environment. This has been mostly studied in regard to children and adolescents.

Enormous amounts of advertising for unhealthy foods are directed at children and adolescents. A major part of this is for fast-food restaurants. These adverts can be very successful in inducing the target group to consume the advertised foods [51,52,53]. Not surprisingly, evidence suggests that this advertising is one more factor linked to obesity [53,54]. 

The advertising of unhealthy foods serves no useful purpose. It makes sense, therefore, for this advertising to be banned, especially when it is targeted toward children and adolescents. As children are commonly watching TV late in the evening, a ban should cover all programs where large numbers of young people are likely to be watching. There is evidence that such bans can be of value in reducing the consumption of unhealthy food [55]. Policies along these lines have been implemented by numerous local and national governments. There is a strong case for banning all advertising for unhealthy foods, including that directed at adults. 

A marketing method used by the food industry is the placement of junk food close to the checkout in supermarkets. This has the obvious intention of boosting sales of these foods. Studies carried out in the UK indicate that the removal of these foods from the area around the checkout reduces sales [56]. This points to another policy that deserves support alongside a ban on the advertising of unhealthy foods.

## 6. Barriers to the Implementation of Government Policies

It is highly predictable that food corporations and their lobbyists will use their considerable resources in order to obstruct the implementation of the type of policies being advocated here [11]. They will almost certainly use a game plan similar to that developed decades ago by the tobacco industry. That industry did everything within its powers to thwart all efforts by health advocates and governments to implement vital policies such as higher taxes on cigarettes and a ban on smoking in places where nonsmokers may be exposed to cigarette smoke. It is therefore probable that it will require much determination and sustained effort before any serious action is taken to deconstruct the obesogenic environment. But the experience of Latin America demonstrates that real progress is possible. The governments of Mexico, Chile, and Brazil have passed laws and implemented policies similar to those advocated here [57]. The goal is to reduce the consumption of unhealthy foods. Policies include limiting the sale of these foods at schools, using taxes, and restricting advertising where children are the target.

## 7. Conclusions

Many aspects of the environment act together to cause obesity. These factors include food composition, food prices, advertising of unhealthy foods, and a lack of physical activity. The combined result is that the environment as a whole is obesogenic. Many interventions have been used in order to counter obesity.

One strategy is health promotion. Interventions typically rely on health education and encouragement in an attempt to persuade the target population to adopt a healthier lifestyle, including improved weight control. These interventions have mostly achieved only limited success. This strategy is very unlikely to have a major impact on the obesity epidemic.

The only strategy that offers a real chance of ending the obesity epidemic is the implementation of policies that neutralize the obesogenic environment. This means radically changing the current environment away from one that results in much of the population following a lifestyle that encourages excessive food intake, especially of ultra-processed foods, and of sedentary behavior. More broadly, we must work to create an environment that fosters a generally healthy lifestyle that leads to significant improvements in population health. This requires many changes in government policies, regulations, and laws.

Several key policies were proposed in this paper, including the following:

1. Schools should implement policies that help prevent excessive weight gain. In particular, foods consumed at schools must be of high nutritional quality.
2. There is a need for diverse policies directed at children and adolescents that go beyond schools and that are likely to reduce the burden of obesity.
3. Healthy foods must be made more affordable than less healthy foods. This is best achieved by adding subsidies to healthier foods and taxes to unhealthy foods.
4. Adverts for unhealthy foods (including fast-food restaurants) should be banned, especially when the target audience is children and adolescents. It makes sense to extend such a ban to a complete ban of advertising unhealthy foods.
5. While health promotion campaigns (i.e., giving advice and encouragement on nutrition and exercise) are considerably less effective than government policy approaches, they are still of much value and should be expanded.

These proposals are far from comprehensive and many other policies need to be formulated.
